# Synchronization, Slippage, and Unbundling of Driven Helical Flagella

**DOI:** 10.1371/journal.pone.0070868

**Published:** 2013-08-19

**Authors:** Shang Yik Reigh, Roland G. Winkler, Gerhard Gompper

**Affiliations:** Theoretical Soft Matter and Biophysics, Institute of Complex Systems and Institute for Advanced Simulation, Forschungszentrum Jülich, Jülich, Germany; German Cancer Research Center, Germany

## Abstract

*Peritrichous* bacteria exploit bundles of helical flagella for propulsion and chemotaxis. Here, changes in the swimming direction (tumbling) are induced by a change of the rotational frequency of some flagella. Employing coarse-grained modeling and simulations, we investigate the dynamical properties of helical flagella bundles driven by mismatched motor torques. Over a broad range of distances between the flagella anchors and applied torque differences, we find a stable bundled state, which is important for a robust directional motion of a bacterium. With increasing torque difference, a phase lag in the flagellar rotations develops, followed by slippage and ultimately unbundling, which sensitively depends on the anchoring distance of neighboring flagella. In the slippage and drift states, the different rotation frequencies of the flagella generate a tilting torque on the bacterial body, which implies a change of the swimming direction as observed experimentally.

## Introduction

Motile bacteria exploit bundles of rotating helical flagellar filaments for propulsion and chemotaxis [1]–[4]. Various arrangements of the flagella on the bacterial cell membrane have developed in the evolutionary process [5]. The flagella of *peritrichous* bacteria self-organize into bundles by (typically) counterclockwise rotation of the flagella motors leading to nearly straight swimming. To change the swimming direction, this “running” phase is interrupted by short periods of “tumbling” [6]–[8]. A specific flagella design [9] implies a distinct tumbling mechanism of a particular bacterium. In *Escherichia coli* bacteria, clockwise rotation of one or several flagella leads to a polymorphic transition, [10]–[14] disintegration of the bundle and bacterial tumbling [1], [15], [16]. In contrast, the flagella of *Rhizobium meliloti* or *Rhizobium lupini* are only capable of limited polymorphic transitions and their motors are unidirectional [9], [17], [18]. These bacteria modulate the rotation speed of individual motors to induce tumbling [9], [17].

The bundling process of bacterial flagella has been studied experimentally [3], [17], [19]–[21], theoretically [22]–[24], and by computer simulations [5], [8], [16]. An essential aspect of bundling is synchronization of the flagella driven by motors of nonuniform strength. Synchronization of flagellar rotation is, aside from bacterial motion, of fundamental importance for a broad range of phenomena in biology [25]–[29], ranging from fluid transport in the respiratory system [30], to embryonic left-right asymmetry [31], and intercellular communication [32]. Thus, a theoretical understanding of flagella synchronization is of paramount importance. Synchronization in fluid systems can be achieved by hydrodynamic interactions as demonstrated by various studies [16], [27], [28], [33]–[40]. In bacterial bundling, steric interactions between the various flagella may also play an important role due to the opposite rotation of the flagella bundle and the cell body [16].

Much less is known about the tumbling mechanism of bacteria in general, and for bacteria such as *R. lupini* or *R. meliloti* with unidirectional motors in particular. In Ref. [17], bundle disintegration has been observed when a flagellar filament slows down or stops. However, tumbling should also be possible without complete disintegration of the bundle, but might be more difficult to observe experimentally.

Here, we study synchronization and unbundling of driven flagella, properties which are of fundamental importance for bacterial tumbling. Of particular interest is the dephasing of flagella rotational frequencies with increasing torque difference between the various flagella. Qualitatively, our results suggest the following classification of the bundle dynamics. At small torque differences, the bundle remains stable with a phase lag between the various flagella. For very large torque differences, the bundle disintegrates and the flagella rotated asynchronous and independently; the phase differences of neighboring helices are drifting. In between, there is an intermittent regime, where phase slippage occurs, i.e., the synchronized rotational motion is interrupted by events, where the flagellum with the larger torque leaves the bundle, rotates faster, and rejoins the bundle. The interval between individual slippages decreases with increasing torque difference and ultimately drifting is obtained. We predict that this slippage or drift leads to a tumbling motion of the bacteria.

## Models

**Figure 1:**
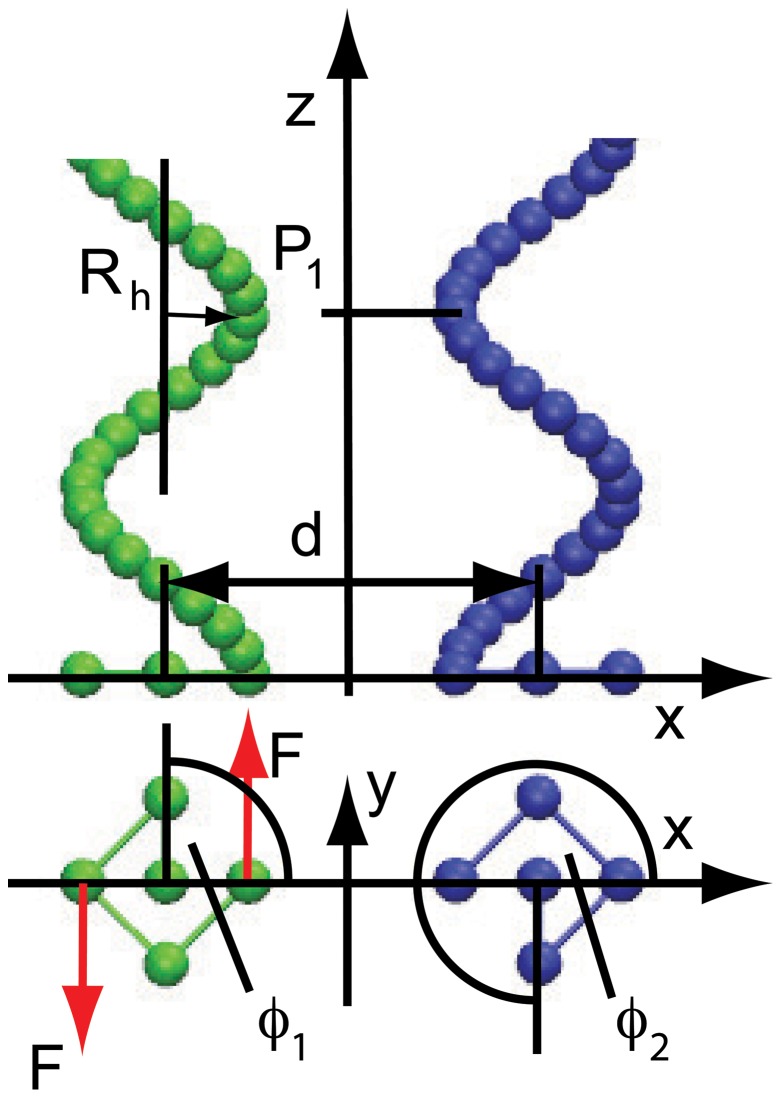
Model of a flagella bundle. The top part shows a side view of two left-handed flagella, with average distance 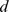, helix radius 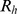, and pitch 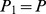. The bottom part shows a top view with the external forces 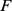 generating a torque and the phase angles 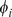.

A hybrid mesoscopic simulation approach is adopted, combining molecular dynamics simulations for the bacterial flagella with the multiparticle collision dynamics (MPC) approach, a mesoscale hydrodynamics simulation technique, for the fluid [41]–[43]. This hybrid method has been successful applied to study the dynamics of various kinds of macromolecular and cellular systems [35], [40], [44], [45]. The bacterial flagellum is represented by a coarse-grained macromolecular system embedded in a MPC fluid with a helical sequence of 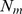 mass points of mass 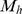 [16] (cf. Fig. 1). These points interact with each other by the following bond, bending, and torsional potentials:
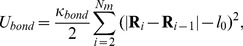
(1)

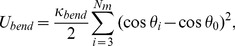
(2)

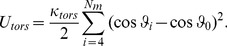
(3)Here, 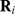 denotes the position of bead 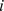, and 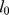, 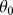, and *φ*_0_ are the equilibrium bond length, bending angle, and torsional angle, respectively. 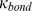, 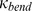, and 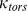 are the bond, bending, and torsional rigidities, respectively. The repulsive and truncated Lennard-Jones potential
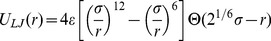
(4)is applied to account for excluded-volume interactions between the mass points of the helices of distance 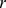, with the size of a point 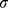, the energy 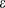 [16], [46], and 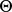 the Heaviside step function. In addition, one central bead and four peripheral beads are added in a plane at the base of the helix (cf. Fig. 1). These five beads are trapped in constraining harmonic potentials of the form,
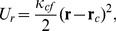
(5)where 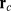 is either the equilibrium position of the central particle or the 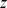-coordinate of the peripheral beads; in the latter case, the 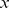
- and 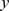-coordinates are unconstrained. Hence, a flagellum is not allowed to perform any translational motion but rotates around a central bead driven by an external torque applied at two opposite peripheral beads as illustrated in Fig. 1. The dynamics of the mass points is described by Newton's equations of motion, which are integrated by the velocity-Verlet algorithm [46]. Symmetrical forces are applied in counterclockwise direction when watched from the distal end, which generate a torque 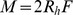 pointing into the positive 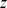-direction, where 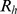 is the helix radius and 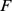 the applied force.

MPC is a particle-based simulation approach, where the fluid is represented by point particles, and naturally comprises thermal fluctuations. The algorithm consists of alternating streaming and collision steps. In the streaming step, the particles move ballistically; in the collision step, the particles are sorted into cubic cells of length 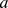 and their relative velocities, with respect to the center-of-mass velocities of each cell, are rotated around a randomly oriented axis by a fix angle 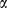 [16], [43]. A constant temperature is maintained locally by velocity rescaling at every collision cell and every collision step [47]. The MPC parameters are chosen as: average fluid particle number per collision cell 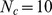, rotation angle 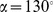, and collision time step 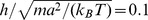, where 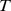 is the temperature, 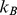 the Boltzmann constant, and 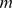 the mass of a fluid particle, which yields the solvent viscosity 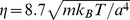 [16], [48]. The parameters for a flagellum are chosen as: mass of a flagellum bead 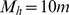, helix radius 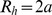, number of beads for a five-turn helix 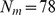, equilibrium bond length 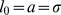, bending angle 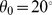, torsional angle *φ* 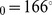, and 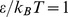. The bond stretching, bond bending, and torsional rigidities are 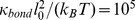, 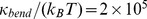, and 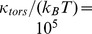, respectively. The constraining-force constant of the motor part is 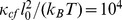. The distribution of the bond stretching, bending, and torsional energies follows the corresponding Boltzmann distributions.

The adopted potential parameters and contour length yield a five-turn helix with the pitch angle 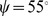. This closely resembles the shape of a flagellum of *R. lupini* in the semi-coiled state [17]. The bending rigidity of a *Salmonella* flagellum has been determined in Ref. [13]. A value of 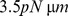 for the bending rigidity was obtained by employing a model with a quadratic Kirchhoff-rod potential for bending and twist, and comparing the theoretical force-extension relation with the respective experimental data. Moreover, the twist rigidity was found to be comparable with the bending rigidity [13]. In order to link the parameters 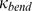 and 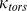 in Eqs. (2) and (3) with the experimental results for the flagellum rigidities, we discretize the continuum bending and torsional energies of the helical wormlike chain model [13], [49] and compare them with the potentials (2) and (3). Using the structural parameters of *R. lupini* [17], we find that our 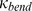 corresponds to an approximately five times larger bending rigidity compared to the experimental value for *Salmonella*. Since it has been argued that the flagellum of *R. lupini* is stiffer than that of *Salmonella* [50], our chosen value is in reasonable agreement with the biologically relevant scales. The ratio between the bending and the torsional rigidities in our model is approximately four. Hence, also the torsional rigidity is on the order of magnitude of the biological scale.

The flagella are placed in a cubic simulation box of side length 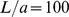 with periodic boundary conditions. When the flagella start to rotate, they set fluid in motion until a stationary (mean) fluid velocity is reached, where the flagella exert no net force on the anchoring plane along its normal. This is equivalent to a free-swimming bacteria with a non-rotating body. In our system, the fluid velocity far away from the flagella corresponds to the swimming velocity of the free swimmer, because the two systems are just related by a Galilean transformation. This point will be discussed in more detail below.

**Figure 2:**
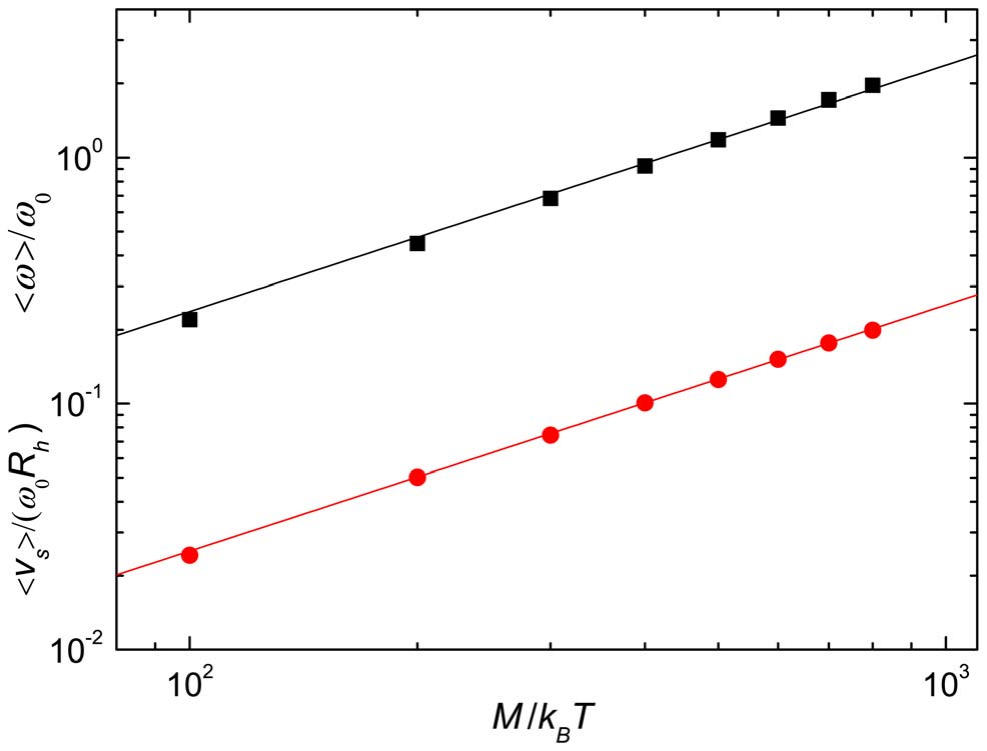
Single flagellum rotation frequency and swimming speed. Mean values of the rotation frequencies (squares) and mean induced fluid velocities (bullets) of a single helix for various applied torques. The lines indicate the linear dependence on the torque.

We have performed several test to validate the selected model using a single flagellum. As expected and shown in Fig. 2, we find a linear relation between the applied torque and the rotation frequency of the helix. Since the helix is fixed at the anchoring point, the fluid acquires a constant mean velocity in the stationary state. This velocity also depends linearly on the applied torque, see Fig. 2. The reference frequency 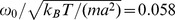 corresponds to the rotation frequency of a bundle of three helices each driven by the torque 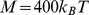 at the distance 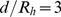. A single helical flagellum exhibits only a few percent smaller rotation frequency for the same torque.

## Results and Discussion

Corresponding to the averaged number of experimentally observed flagella for *R. lupini* [17], we study a system of three helices with their anchoring points fixed on an equilateral triangle of side length 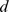. Figure 1 illustrates the initial parallel alignment for two helices and the phase angles 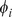 (in general, 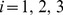). The initial angles of the three helices are 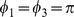 and 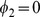. To study the bundle response to differences in the applied torques 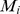 at the various helices, we utilize the fixed torque 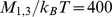 of helices 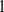 and 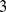 and vary that of helix 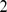 in the range 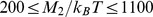. The experimentally measured torques for flagella of *E. coli* bacteria are in the range from 1300 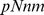 to 4600 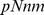, i.e., 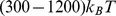 [7], [51]–[53]. Thus, our simulated torques cover approximately the same range at room temperature.

### Flagella bundle stability

**Figure 3:**
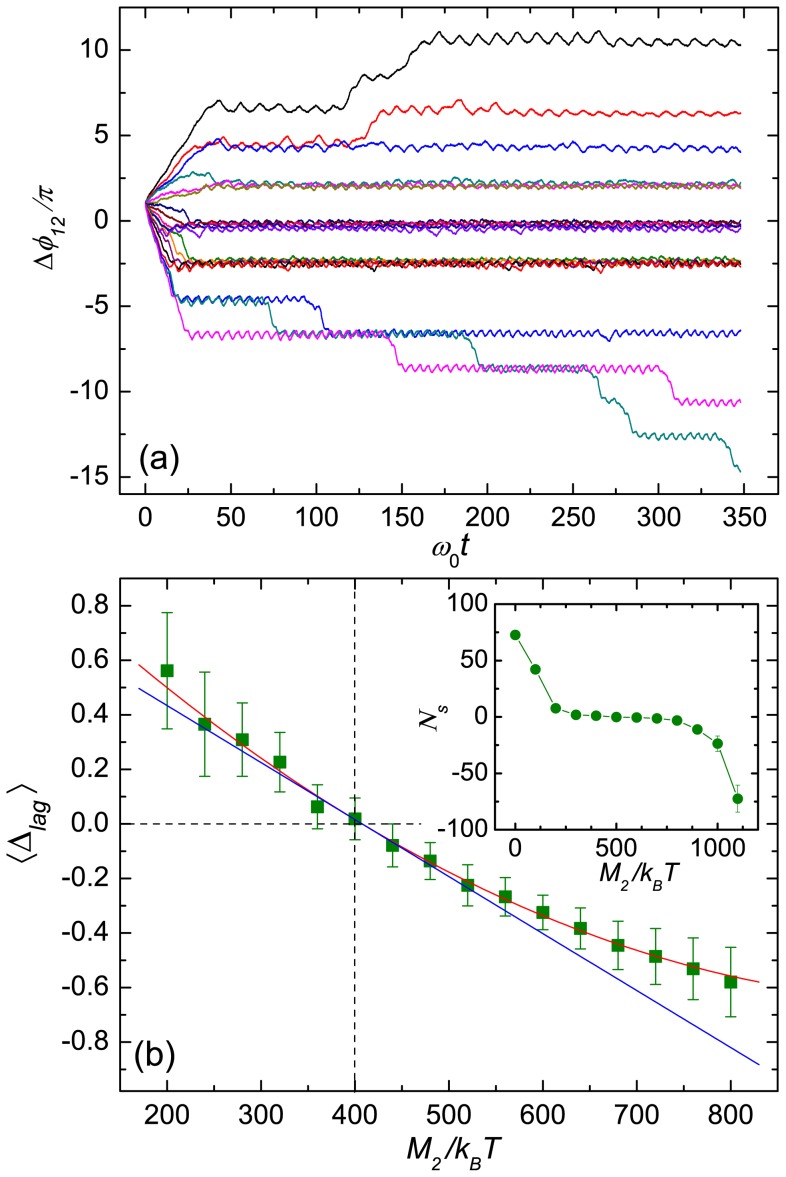
Phase slip, lag, and drift. (a) Phase angle difference 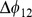 as a function of time for various applied torques on helix 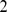 and the distance 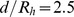. The torque 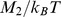 is changed from 200 (top) to 920 (bottom) with an increment of 40; the constant torque is 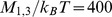. (b) Average phase lag 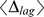 as a function of the torque 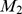. The red line indicates the fit 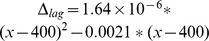, where 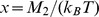. The blue line is the tangent at 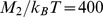. The inset provides an approximate measure of the number of occurring slips during the time interval 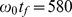 as a function of the applied moment 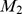.

Figure 3(a) displays the phase-angle difference 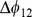 (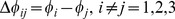) as a function of time for various torques 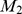. The time scale is normalized by the reference angular velocity 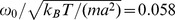 of a bundle of three helices, as already introduced in the previous section. For torques in the range 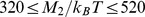, the helices form bundles and exhibit a phase-locked synchronized rotational dynamics with the same average angular frequency after a short time. As discussed in Ref. [16], synchronization is achieved by hydrodynamic and steric interactions between the various helices. When the torque difference 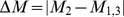 exceeds a critical value, phase slips occur. Here, flagella with the larger torque leave the bundle and perform one additional rotation and then rejoin the bundle; this corresponds to a change in phase difference by 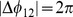. With increasing 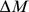, the frequency of phase slips increases and ultimately the drift state is reached, where 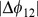 increases linearly in time. The appearance of slips is governed by the interplay of excluded volume interactions and elastic deformations of the flagella as well as thermal fluctuations.

In the synchronized and bundled state, the helices exhibit a phase lag 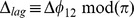, which depends on the torque difference. The obtained phase lags exhibit considerable fluctuations around the average. The magnitude of the average 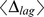 increases with increasing torque difference. As shown in Fig. 3(b) by a quadratic fit function, 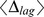 is asymmetric with respect to 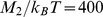 with a slower variation in the region of large 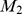. This “asymmetry” appears due to the increase of the bundle rotational frequency with increasing 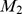. The larger frequencies imply stronger flows and therefore stronger hydrodynamic interactions and tighter bundles.

The inset of Fig. 3 displays the quantity 
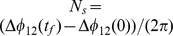
 for the fixed time interval 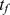, which is an approximate measure of the number of occurring slips. For momenta 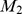 in the vicinity of 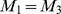, phase slips are rare. However, we find a strong increase of slippage, when the torque difference 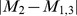 exceeds a threshold.

**Figure 4:**
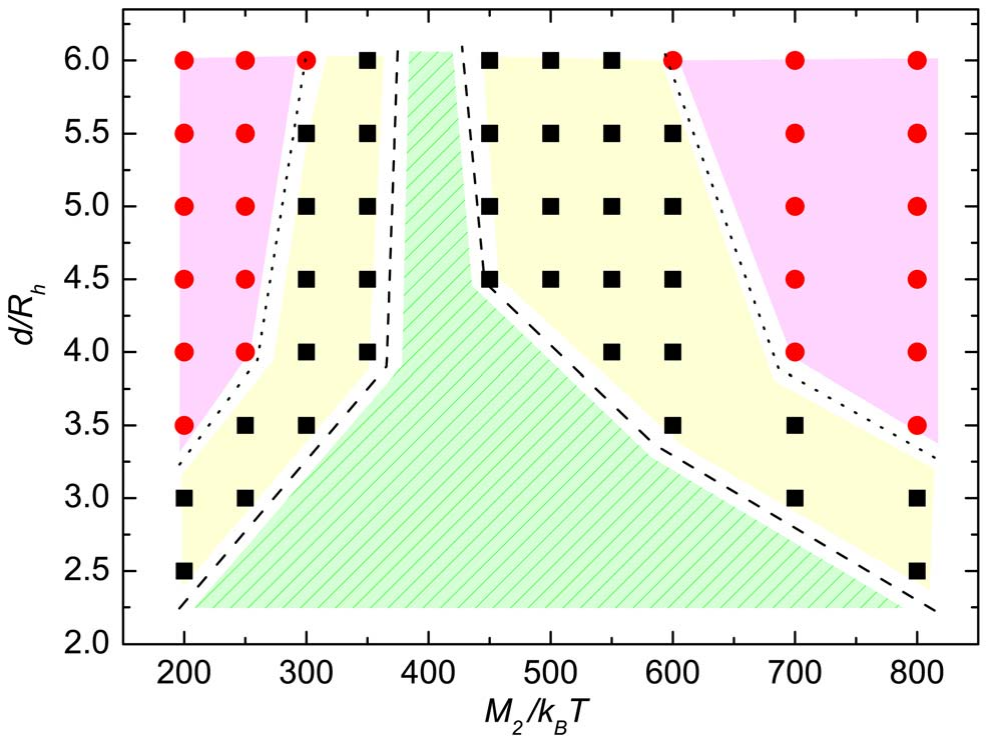
Phase diagram of bundle integrity. Phase diagram indicating stable bundles (shaded), intermittent slippage (squares), and drift states (bullets) for various flagella distances and torques on helix 2. The torques on helices 1 and 3 are 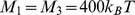.

Data for the bundling, slippage, and drifting states for different torques and helix separations can be combined in a dynamic phase diagram, as shown in Fig. 4. Interestingly, there is a broad range of distances and torques, where stable bundles are obtained with no slips over the total considered time interval 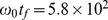. The bundles are evidently rather robust for 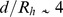, and are able to sustain considerable torque differences. The asymmetry of the phase diagram with respect to the reference torque 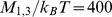 is a consequence of the stronger hydrodynamic interactions at larger torques (cf. Fig. 3(b)). The stable bundle regime is bounded by the intermittent slippage regime, where 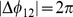 for each individual slippage event. Since this part of the diagram is also broad, the model system yields stable or nearly stable bundles over a wide range of torques for all the considered distances. For even larger distances and torque differences phase-drift appears, where 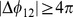 before a loose bundle might be reformed. Naturally, a clear distinction between the various regimes is difficult and the change from one to the other is gradual rather than abrupt.

The phase diagram of Fig. 4 highlights a strong phase-locking for bundles of flexible helical flagella. Even for distances as large as 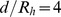, phase locking occurs for torque differences as large as 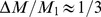. This can be compared with the synchronization of rigid, three-arm colloidal micro-rotators discussed in Ref. [38], which can tolerate only torque differences of the order of 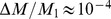 at much smaller distances of 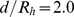 to 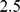. This largely enhanced stability is due to the flexibility of the bacterial flagella, which allows them to wrap around each other. A high stability of the bundled state is very important for the directional motion of a bacterium because of the substantial noise inherent in biological systems, either originating from internal sources like variations in motor activities, or from external sources such as other swimming bacteria.

### Intra-bundle distances

**Figure 5:**
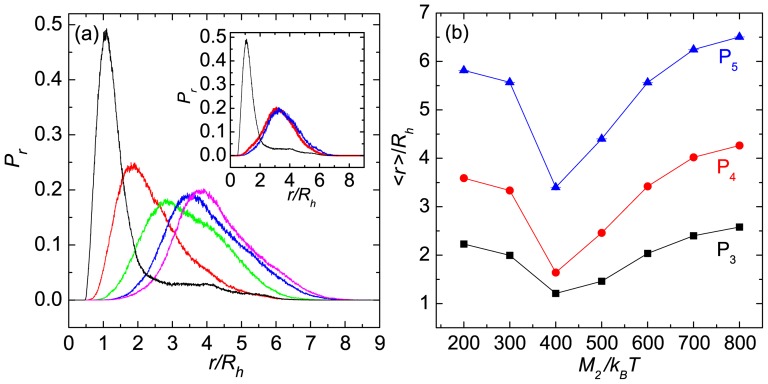
Bead distance distributions and mean distances. (a) Normalized bead-distance distribution functions 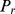 between helices at 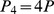, 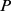 is the helical pitch (cf. Fig. 1), for the torque 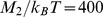 (black), 500 (red), 600 (green), 700 (blue), and 800 (magenta) with 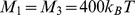. The distance between anchored helix ends is fixed at 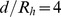. The inset shows the distribution functions for 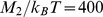 (black), 300 (red), and 200 (blue). (b) Average bead distances between the helices at 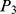 (black), 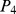 (red), and 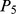 (blue) (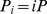) as a function of the torque 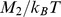.

**Figure 6:**
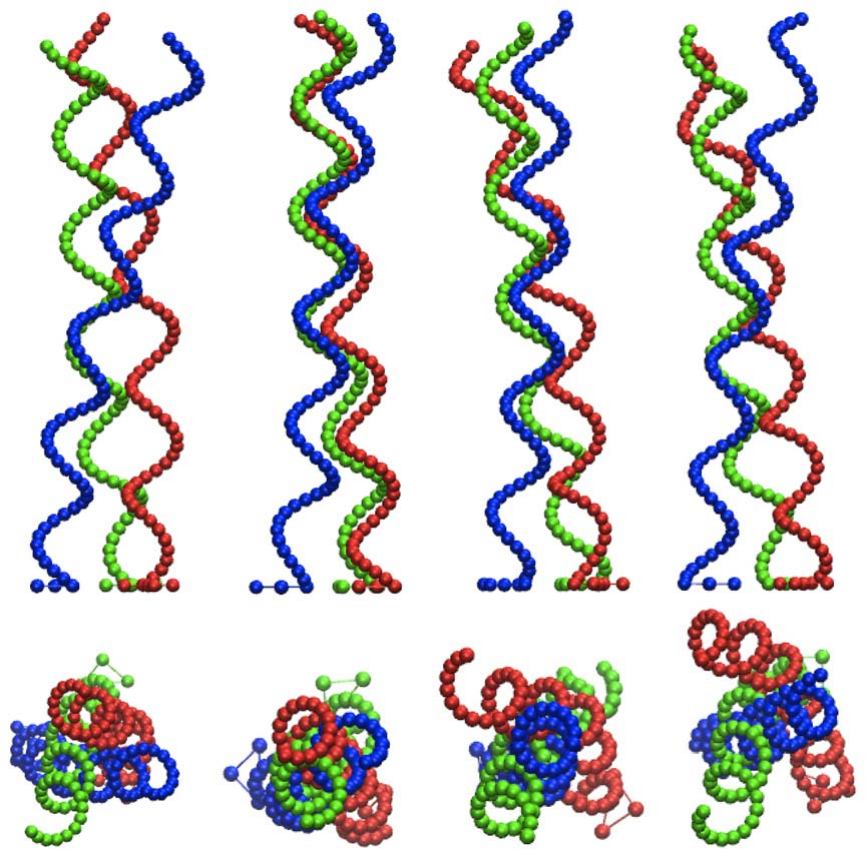
Snapshots of helices for various torque differences. Snapshots of side (top) and top (bottom) views for the torque 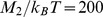, 400, 600, and 800 (from left to right) at 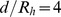. See also videos S1 8 and S2 8 for 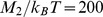 and 800, respectively.

Slippage or drift of individual helices leads to partial or full flagella unbundling, which is reflected in the distribution 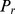 of distances between the equivalent beads of the various helices. Figure 5(a) provides an example for the distributions at 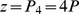, where 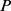 is the pitch, and 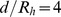. For equal torques, we obtain a tight bundle with a narrow distribution and a maximum at the single monomer diameter. As the torque difference increases, the peak maximum shifts to large distances and the width broadens. The inset of Fig. 5 shows distance distributions for 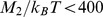. Here, the maximum of 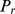 also moves to large distances with increasing torque difference, but saturation seems to be reached already at rather small 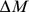, much smaller than for 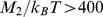. The mean values of the distances between helical beads are shown in Fig. 5(b) as a function of torque 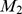 at the points 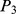, 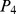, and 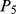 along the bundle. Evidently, the helices form a tight bundle for equal torques (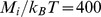). As the torque difference 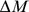 increases, the bundle disintegrates and the helices move apart. The snapshots of Fig. 6 illustrate the bundle geometry for various 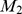 (see also videos S1 and S2). Interestingly, the bundle does not completely disintegrate. The hydrodynamic interactions continuously force the helices back into a bundle. Nevertheless, generation of a large torque difference is an effective strategy to impose helix unbundling.

### Forces on the bacterial body by flagella rotation

**Figure 7:**
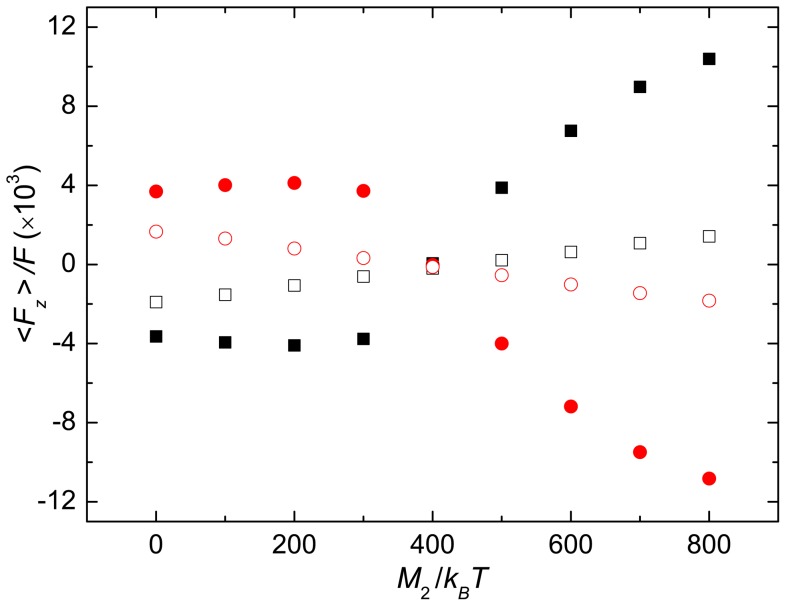
Average forces on bacteria body. Average forces per monomer on the anchoring plane of three helices as a function of the torque 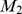 for 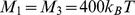 at 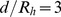. The bullets indicated the forces by helix two and the solid squares those by helix one and three. The circles and open squares are the contributions by the corresponding hydrodynamics forces for unbundled helices.

In the stationary state, the flagella exert no net force on the anchoring plane parallel to its normal for equal torques. Fluid is pumped by the flagella until an stationary fluid velocity is reached and the system is force free. For equal torques this applies also to the individual flagella. However, the situation is changed for non-zero torque differences, where the various helices exert different forces on the anchoring plane. Figure 7 shows average forces for various torque difference. For torques 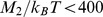, helix 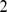 experiences a positive force 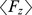, i.e., pointing along the positive 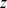-direction, and the other two experience a negative force, i.e., pointing in negative 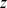-direction. The force orientation is reverse for 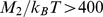. However, the sum of the corresponding forces in the 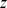-direction still vanishes, as expected, i.e., the total system is force free.

These forces are of various origin, depending on the bundling state. When the helices are bundled and show a phase lag, particularly for 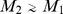, they mainly appear due to excluded-volume interactions between the beads. The calculation of the total Lennard-Jones force between the various helices approximately agrees with the total force 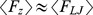. In contrast, for unbundled helices, i.e., large torque differences, hydrodynamic interactions yield a significant contribution to the forces. In case of the slower rotating helix, the fast rotating helices create a fluid flow which drags the other helix along, since the mean fluid velocity originating from their rotation is faster than the fluid velocity due to the rotation of the slow helix. The situation is reverted for a fast rotating helix. Here, the correspondingly fast moving fluid is slowed down by the other helices, which adds an additional drag to the fast moving helix.

Since it is difficult to estimate the contribution of hydrodynamics on the total force quantitatively in the time-dependent slippage state, we confine the individual beads of the three helices in harmonic potentials, such that the helices remain parallel aligned along the 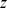-axis. Explicitly, the potentials are
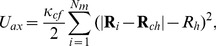
(6)where 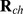 has the same 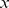
- and 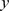-component as the central bead 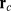 and the same 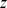-component as 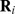 initially [16]. Thus, in this special case, the helices rotate freely and do not form a bundle. Now, the force exerted on the anchoring plane is of hydrodynamic origin only. As shown in Fig. 7, this hydrodynamic force is comparable to that by excluded-volume interactions and the sum is very close to the total force obtained for free helix bundles for 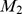 significantly smaller than 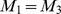. We expect even more significant hydrodynamic contributions for bundles in the slippage and drift states.

For flagellated bacteria, different torques on the various flagella should be the rule rather than the exception. Based in our simulation results, we expect that the forces created by torque differences in the bundled state lead to a wobbling motion, because both, the body and the helices, rotate. In the slipping state, the larger forces should give rise to a pronounced helical swimming trajectory of a bacterium. In the case of unbundling, either by reverse rotation or stopping of a motor, the rotational motion of the body is strongly reduced, and a large change in body orientation should be generated. Hence, the different dynamical states of flagella (cf. Fig. 4)) imply different swimming modes. A sampling of a larger spatial area is achieved by helical paths in the bundled and slippage state, whereas major directional changes are achieved in the unbundled state.

**Figure 8:**
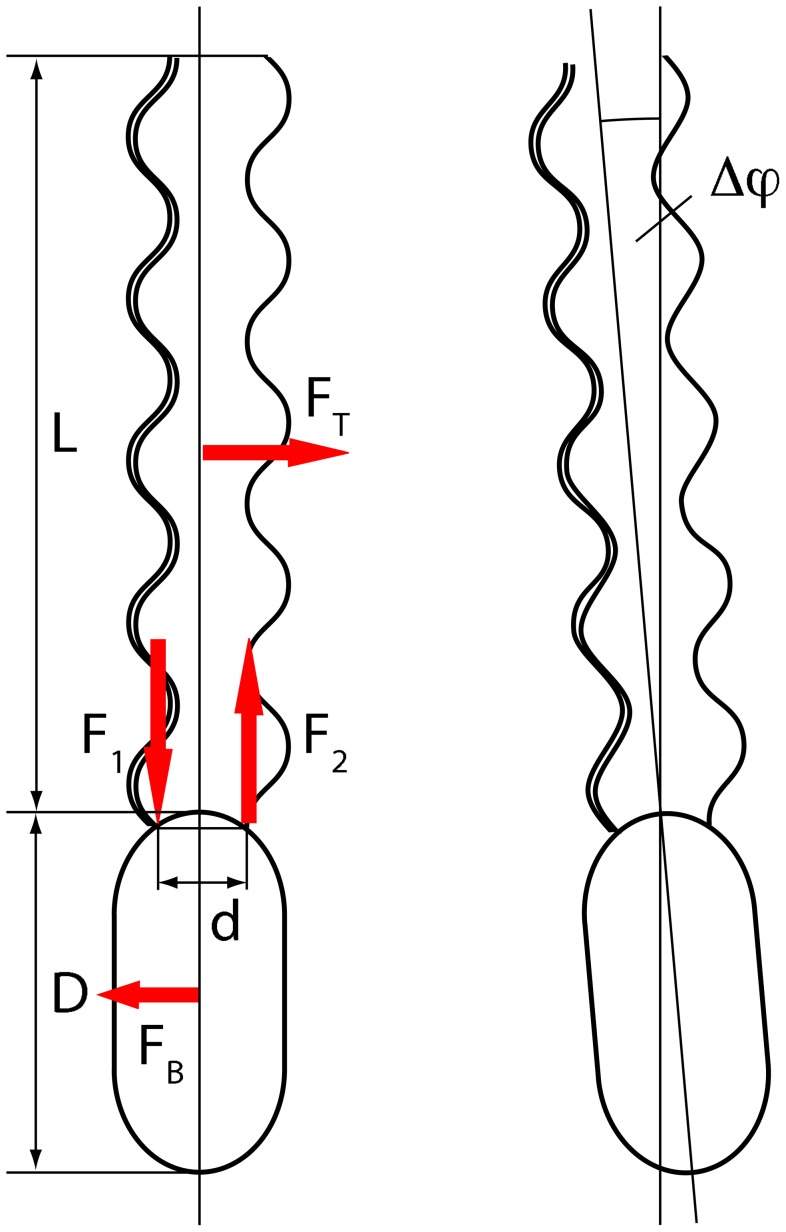
Bacterial reorientation. Illustration of the forces and torques on a bacterium and the estimated change in orientation 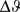. 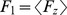 and 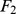 are the excess forces after the mean driving force has been subtracted. The induced torque rotates the whole structure, which gives rise to the drag forces 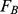 on the cell body and 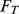 on the tail.

An estimation of the change in the bacterial orientation 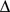 *φ* during the time interval 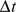 by the forces 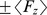 can be obtained from the following simple arguments. The torque due to friction with the fluid of the bundle and cell body is 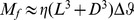 *φ* 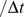 [54], where 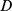 is the length of the cell body and 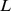 the length of the bundle (cf. Fig. 8). This torque is balanced by the torque 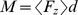. Hence, for one period of the bundle rotation, i.e., 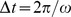, we find 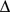 *φ* 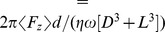, with the bundle rotation frequency 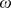. For values obtained from the simulations, 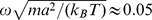, 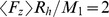, and the estimated parameters of the bacterial geometry of *R. lupini* [17] of 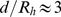, 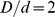, and 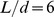, we find 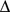 *φ* 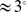 for a single flagellar rotation. This adds up to approximately 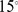 after about five rotations, a value in reasonable agreement with the rotation angle for *R. lupini* extractable from the images in Fig. 3 of Ref. [17].

### Summary

We have been investigating the properties of rotating bacteria flagella, where individual flagella are driven by motor torques of different strengths. In particular, we have focused on the stability of the flagella bundle, an aspect which is important for both, the swimming of an bacterium and its tumbling by partial degradation of the bundle. Our coarse-grained mesoscale simulations demonstrate that hydrodynamic interactions between flagella, short-range volume exclusion which prevents the intersection of flagella on contact, and the flagellar flexibility which allows for partial wrapping of flagella around each other, are all essential ingredients in the bacterial tumbling process. The interplay of these physical mechanisms not only cause synchronization and bundling of rotating flagella, but also guarantee the robustness of bundle formation in the presence of small differences in motor torques, which is essential for directed bacterial locomotion. For larger torque differences, the competition of hydrodynamic interactions with volume exclusion and elastic deformation of the flagellum leads to slippage and drift, which implies a reorientation of the swimming direction. It would be interesting to search for flagellar slippage in motile bacteria experimentally.

## Supporting Information

Video S1
**Flagella bundle of three helices with the motor torques** 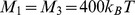 **(green, blue) and** 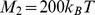 **(red).**
(MPG)

Video S2
**Flagella bundle of three helices with the motor torques** 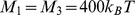 **(green, blue) and** 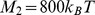 **(red).**
(MPG)

## References

[1] Berg HC, Brown DA (1972) Chemotaxis in *Escherichia coli* analysed by three-dimensional tracking. Nature 239: 500–504. 4563019 10.1038/239500a0

[2] Berg HC, Anderson RA (1973) Bacteria swim by rotating their flagella filaments. Nature 245: 380–382. 4593496 10.1038/245380a0

[3] Berg HC (2004) E. Coli in Motion. New York: Springer.

[4] Taylor GI (1951) Analysis of the swimming of microscopic organisms. Proc Roy Soc A 209: 447–461.

[5] Janssen PJA, Graham MD (2011) Coexistence of tight and loose bundled states in a model of bacterial flagella dynamics. Phys Rev E 84: 011910. 10.1103/PhysRevE.84.011910 21867216

[6] Turner L, Ryu WS, Berg HC (2000) Real-time imaging of fluorescent flagellar filaments. J Bacteriol 182: 2793–2801. 10781548 10.1128/jb.182.10.2793-2801.2000 PMC101988

[7] Darnton NC, Turner L, Rojevsky S, Berg HC (2007) On torque and tumbling in swimming *Escherichia coli* . J Bacteriol 189: 1756–1764. 17189361 10.1128/JB.01501-06 PMC1855780

[8] Watari N, Larson RG (2010) The hydrodynamics of a run-and-tumble bacterium propelled by polymorphic helical flagella. Biophys J 98: 12–17. 20074512 10.1016/j.bpj.2009.09.044 PMC2800969

[9] Platzer J, Sterr W, Hausmann M, Schmitt R (1997) Three genes of a motility operon and their role in flagellar rotary speed variation in *Rhizobium meliloti* . J Bacteriol 179: 6391–6399. 9335288 10.1128/jb.179.20.6391-6399.1997 PMC179555

[10] Calladine CR (1975) Construction of bacterial flagella. Nature 225: 121–124. 10.1038/255121a0 1128677

[11] Calladine CR (1978) Change of waveform in bacterial flagella: the role of mechanics at the molecular level. J Mol Biol 118: 457–479.

[12] Macnab RM, Ornston MK (1977) Normal-to-curly flagella transitions and their role in bacterial tumbling. stabilization of an alternative quaternary structure by mechanical force. J Mol Biol 112: 1–30. 328893 10.1016/s0022-2836(77)80153-8

[13] Darnton NC, Berg HC (2007) Force-extension measurements on bacterial flagella: triggering polymorphic transformations. Biophys J 92: 2230–2236. 17172309 10.1529/biophysj.106.094037 PMC1861800

[14] Wada H, Netz RR (2008) Discrete elastic model for stretching-induced flagellar polymorphs. Europhys Lett 82: 28001.

[15] Xie L, Altindal T, Chattopadhyay S, Wu XL (2011) Bacterial flagellum as a propeller an as a rudder for efficient chemotaxis. Proc Natl Acad Sci USA 108: 2246–2251. 21205908 10.1073/pnas.1011953108 PMC3038696

[16] Reigh SY, Winkler RG, Gompper G (2012) Synchronization and bundling of anchored bacterial flagella. Soft Matter 8: 4363–4372.

[17] Scharf B (2002) Real-time imaging of fluorescent flagellar filaments of *Rhizobium lupini* h13-3: flagellar rotation and ph-induced polymorphic transitions. J Bacteriol 184: 5979–5986. 12374832 10.1128/JB.184.21.5979-5986.2002 PMC135403

[18] Schmitt R (2002) Sinorhizobial chemotaxis: a departure from the enterobacterial paradigm. Microbiology 148: 627–631. 11882696 10.1099/00221287-148-3-627

[19] Macnab RM (1977) Bacterial flagella rotating in bundles: a study in helical geometry. Proc Natl Acad Sci USA 74: 221–225. 264676 10.1073/pnas.74.1.221 PMC393230

[20] Kim M, Bird JC, Parys AJV, Breuer KS, Powers TR (2003) A macroscopic scale model of bacterial flagellar bundling. Proc Natl Acad Sci USA 100: 15481–15485. 14671319 10.1073/pnas.2633596100 PMC307593

[21] Qian B, Jiang H, Gagnon DA, Breuer KS, Powers TR (2009) Minimal model for synchronization induced by hydrodynamic interactions. Phys Rev E 80: 061919. 10.1103/PhysRevE.80.061919 20365202

[22] Reichert M, Stark H (2005) Synchronization of rotating helices by hydrodynamic interactions. Eur Phys J E 17: 493–500. 16096696 10.1140/epje/i2004-10152-7

[23] Flores H, Lobaton E, Mendez-Diez S, Tlupova S, Cortez R (2005) A study of bacterial flagellar bundling. Bull Math Biol 67: 137–168. 15691543 10.1016/j.bulm.2004.06.006

[24] Elfring GJ, Lauga E (2009) Hydrodynamic phase locking of swimming microorganisms. Phys Rev Lett 103: 088101. 19792766 10.1103/PhysRevLett.103.088101

[25] Polin M, Tuval I, Drescher K, Gollub JP, Goldstein RE (2009) *Chlamydomonas* swims with two “gears” in a eukaryotic version of run-and-tumble locomotion. Science 325: 487–490. 19628868 10.1126/science.1172667

[26] Stocker R, Durham WM (2009) Tumbling for stealth? Science 325: 400–402. 19628846 10.1126/science.1177269

[27] Goldstein RE, Polin M, Tuval I (2009) Noise and synchronization in pairs of beating eukaryotic flagella. Phys Rev Lett 103: 168103. 19905728 10.1103/PhysRevLett.103.168103

[28] Golestanian R, Yeomans JM, Uchida N (2011) Hydrodynamic synchronization at low reynolds number. Soft Matter 7: 30743082.

[29] Friedrich BM, Jülicher F (2012) Flagellar synchronization independent of hydrodynamic interactions. Phys Rev Lett 109: 138102. 23030122 10.1103/PhysRevLett.109.138102

[30] Afzelius BA (1976) A human syndrome caused by immotile cilia. Science 193: 317–319. 1084576 10.1126/science.1084576

[31] Cartwright JHE, Piro O, Tuval I (2004) Fluid-dynamical basis of the embryonic development of left-right asymmetry in vertebrates. Proc Natl Acad Sci USA 101: 7234–7239. 15118088 10.1073/pnas.0402001101 PMC409902

[32] Wang Q, Pan J, Snell WJ (2006) Intraflagellar transport particles participate directly in ciliumgenerated signaling in *Chlamydomonas* . Cell 125: 549–562. 16678098 10.1016/j.cell.2006.02.044

[33] Kim M, Powers TR (2004) Hydrodynamic interactions between rotating helices. Phys Rev E 69: 061910. 10.1103/PhysRevE.69.061910 15244620

[34] Kim YW, Netz RR (2006) Pumping fluids with periodically beating grafted elastic filaments. Phys Rev Lett 21: 158101. 10.1103/PhysRevLett.96.158101 16712201

[35] Yang Y, Elgeti J, Gompper G (2008) Cooperation of sperm in two dimensions: synchronization, attraction, and aggregation through hydrodynamic interactions. Phys Rev E 78: 061903. 10.1103/PhysRevE.78.061903 19256864

[36] Uchida N, Golestanian R (2010) Synchronization and collective dynamics in a carpet of microfluidic rotors. Phys Rev Lett 104: 178103. 20482146 10.1103/PhysRevLett.104.178103

[37] Kotar J, Leoni M, Bassetti B, Lagomarsino MC, Cicuta P (2010) Hydrodynamic synchronization of colloidal oscillators. Proc Natl Acad Sci USA 107: 7669–7673. 20385848 10.1073/pnas.0912455107 PMC2867893

[38] Di Leonardo R, Búzás A, Kelemen L, Vizsnyiczai G, Oroszi L, et al (2012) Hydrodynamic synchronization of light driven microrotors. Phys Rev Lett 109: 034104. 22861857 10.1103/PhysRevLett.109.034104

[39] Uchida N, Golestanian R (2011) Generic conditions for hydrodynamic synchronization. Phys Rev Lett 106: 058104. 21405441 10.1103/PhysRevLett.106.058104

[40] Elgeti J, Gompper G (2013) Emergence of metachronal waves in cilia arrays. Proc Natl Acad Sci USA 110: 4470–4475. 23487771 10.1073/pnas.1218869110 PMC3607033

[41] Malevanets A, Kapral R (1999) Mesoscopic model for solvent dynamics. J Chem Phys 110: 8605–8613.

[42] Kapral R (2008) Multiparticle collision dynamics: simulation of complex systems on mesoscales. Adv Chem Phys 140: 89–146.

[43] Gompper G, Ihle T, Kroll DM, Winkler RG (2009) Multi-particle collision dynamics: a particlebased mesoscale simulation approach to the hydrodynamics of complex fluids. Adv Polym Sci 221: 1–87.

[44] Mussawisade K, Ripoll M, Winkler RG, Gompper G (2005) Dynamics of polymers in a particlebased mesoscopic solvent. J Chem Phys 123: 144905. 16238422 10.1063/1.2041527

[45] Huang CC, Winkler RG, Sutmann G, Gompper G (2010) Semidilute polymer solutions at equilibrium and under shear flow. Macromolecules 43: 10107.

[46] Allen MP (1987) Computer Simulation in Liquids. Clarendon: Oxford.

[47] Huang CC, Chatterji A, Sutmann G, Gompper G, Winkler RG (2010) Cell-level canonical sampling by velocity scaling for multiparticle collision dynamics simulations. J Comput Phys 229: 168–177.

[48] Ripoll M, Mussawisade K, Winkler RG, Gompper G (2005) Dynamic regimes of fluids simulated by multiparticle-collision dynamics. Phys Rev E 72: 016701. 10.1103/PhysRevE.72.016701 16090128

[49] Liu Y, Pérez T, Li W, Gunton JD, Green A (2011) Statistical mechanics of the helical wormlike chain model. J Chem Phys 134: 065107. 21322740 10.1063/1.3548885

[50] Götz R, Schmitt R (1987) Rhizobium meliloti swims by unidirectional, intermittent rotation of right-handed flagellar helices. J Bacteriol 169: 3146. 3597320 10.1128/jb.169.7.3146-3150.1987 PMC212363

[51] Berg HC (2003) The rotary motor of bacterial flagella. Annu Rev Biochem 72: 19–54. 12500982 10.1146/annurev.biochem.72.121801.161737

[52] Berry RM, Berg HC (1997) Absence of a barrier to backwards rotation of the bacterial flagellar motor demonstrated with optical tweezers. Proc Natl Acad Sci USA 94: 14433–14437. 9405630 10.1073/pnas.94.26.14433 PMC25012

[53] Reid SW, Leake MC, Chandler JH, Lo C, Armitage JP, et al (2007) The maximum number of torque-generating units in the flagellar motor of *Escherichia coli* is at least 11. Proc Natl Acad Sci USA 103: 8066–8071. 10.1073/pnas.0509932103 PMC1472430 16698936

[54] Doi M, Edwards SF (1986) The Theory of Polymer Dynamics. Oxford: Clarendon Press.

